# Aberrant Production of Th1/Th2/Th17-Related Cytokines in Serum of C57BL/6 Mice after Short-Term Formaldehyde Exposure

**DOI:** 10.3390/ijerph111010036

**Published:** 2014-09-26

**Authors:** Haiyan Wei, Kehong Tan, Rongli Sun, Lihong Yin, Juan Zhang, Yuepu Pu

**Affiliations:** Key Laboratory of Environmental Medicine Engineering of Ministry of Education, School of Public Health, Southeast University, Nanjing 210009, China; E-Mails: why_314614@163.com (H.W.); kehom123@126.com (K.T.); sunrongli5318@gmail.com (R.S.); lhyin@seu.edu.cn (L.Y.)

**Keywords:** formaldehyde, cytokine, cytometric bead array, flow cytometer, serum

## Abstract

Previous studies have shown that formaldehyde (FA) could cause immunotoxicity by changing the number of T lymphocytes and that cytokines play a pivotal role in the regulation of T lymphocytes. However, the previously used cytokine detection methods are difficult to use in the measurement of several cytokines in a small amount of sample for one test. Therefore, the cytometric bead array (CBA) technique was used. CBA showed better analytical efficiency and sensitivity than the previous methods. C57BL/6 mice were exposed to the control (normal saline), low FA concentration (0.5 mg/kg), and high FA concentration (2 mg/kg) for 1 week or 1 month. The contents of cytokines, including Th1-related cytokines (IL-2, IFN-γ, and tumor necrosis factor), Th2-related cytokines (IL-4, IL-6, and IL-10), and Th17-related cytokines (IL-17A), were measured by using the BD FACS Canto II Flow Cytometer and analyzed by FCAP Array**^TM^** Software. Th1/Th2/Th17-related cytokines showed a slightly decreasing trend after low FA exposure. Conversely, a significantly increasing trend was found after high FA exposure. Th1/Th2/Th17-related cytokines all serve important functions in the immune reactions in mice after FA exposure.

## 1. Introduction

Formaldehyde (FA) is commonly used in both industrial and residential environments. FA has widespread exposure and health effects. Thus, studying the physiological changes of various systems after FA exposure is important. Apart from its contact irritation symptoms, affecting the skin, eyes, and the upper and lower respiratory tract, numerous studies have focused on the immunotoxicity and carcinogenicity of FA. Previous studies showed the association between FA exposure and asthma [1,2]. Also, FA exposure may result in immunosuppression by decreasing the CD8^+ ^effector memory T cells [3]. Additionally, FA was classified as a carcinogen (Group 1) for both human and experimental animals, according to the International Agency for Research on Cancer (IARC) [4]. Recently, some epidemiological studies [5,6,7,8] reported a causal association between occupational exposure to FA and hematological tumors, especially myeloid leukemia.

Various studies were conducted to clarify the possible toxicity mechanism of FA. For instance, a significant increase of the CD4- and CD8-positive T lymphocytes was associated with the exposure of adult rats to FA (6 ppm) for 8 h per day for 6 weeks [9]. Moreover, the absolute numbers and percentages of T lymphocytes increased significantly in FA-exposed workers compared with the controls. The mean work duration of FA-exposed workers was 7.3 ± 0.8 years (in the range 0.33 years to 30 years). The mean level of FA exposure (TWA_8h_) was 0.20 ± 0.06 ppm (in the range 0.10 ppm to 0.33 ppm) [10]. However, Zhang *et al.* reported that a significantly lower count of the total number of lymphocytes was found in workers who were exposed to FA at an average level of 1.28 ppm for at least 1 year compared with unexposed controls [11]. Subsequently, Zhang *et al.* investigated each T cell lymphocyte subset and found that counts of regulatory T cells and CD8^+^ effect or memory T cells decreased in FA-exposed workers [3]. Thus, we inferred that these studies showed conflicting FA effects on T lymphocytes because of the different FA exposure levels and times. Overall, results of previous studies suggest that FA may cause an adverse effect on T lymphocyte and may potentially affect the immune system or the hematopoietic system.

T lymphocytes are important for the immune response to exogenous compounds. Generally, T lymphocytes differentiate into T helper 1 (Th1), T helper 2 (Th2), T helper 17 (Th17), or regulatory T cells (Treg) by producing corresponding effector cytokines [12]. Cytokines play a pivotal role in inducing cell-mediated immunity and humoral immunity; thus, the effect of FA exposure on the expression levels of Th1/Th2/Th17-related cytokines needs to be investigated [13]. ELISA or mRNA expression techniques, which were used in previous studies, are difficult to use for measuring several cytokines in a small amount of samples for one test. Considering these difficulties, we applied the cytometric bead array (CBA) (Becton Dickinson, San Diego, CA, USA) technique, which showed higher analytical efficiency and sensitivity than the abovementioned methods [14,15]. Recently, we observed the increasing number of *in vivo* experiments demonstrating a clear association between FA exposure and cytokines [13,16,17,18,19,20]. Thrasher *et al.* showed that activated cell-mediated immunity was associated with long-term FA inhalation in humans. In addition, a significant increase in IL-2 level was observed [20]. The production of Th1/Th2/Th17-related cytokines in skin was significantly increased after inhaling a low concentration of FA combined with topical house dust mite stimulation [19]. However, Ohtsuka *et al.* showed an adverse result, as follows: both the mRNAs of Th1-related cytokines (IFN-γ and IL-2) and Th2-related cytokines (IL-4 and IL-5) showed the tendency to be depressed in BN and F344 rats after inhaling 1% FA aerosol for 5 d [13]. Moreover, the production of cytokines after FA exposure was investigated in *in vitro* studies besides *in vivo* studies [21,22,23,24]. On average, a 16.9-fold increase of the inflammatory response protein IL-8 was observed in human lung epithelial cells after exposure to FA at 1 ppm for 4 h [24]. However, IL-6 production remained unchanged after exposure to FA at 200 μg/m^3 ^compared with air exposure [23]. Therefore, the effect of FA exposure on the secretion of Th1/Th2/Th17-related cytokines in serum remains controversial.

We hypothesized that Th1/Th2/Th17-related cytokines may play an important role in the regulation of FA exposure. We aimed to investigate the production of cytokines in the serum of C57BL/6 mice after short-term FA exposure (1 week or 1 month).

## 2. Materials and Methods

### 2.1. Animals

Thirty-six male C57BL/6 mice aged 6 weeks old to 7 weeks old were purchased from Beijing Wei Tong Li Hua Laboratory Animal Co., Ltd. (Beijing, China) The mice were housed in a specific pathogen free animal house at a temperature of 22 ± 2 °C and a relative humidity of 55 ± 5% with a 12 h/12 h light/dark cycle. The animals received certified standard diet and drinking water *ad libitum*. The mice were randomly assigned to six groups (six animals/group) after one week of acclimatization, with no significant differences in mean weight among the groups. All animals’ experiments were carried out in strict accordance with the recommendations of the Guide for the Care and Use of Laboratory Animals of the State Committee of Science and Technology of the People’s Republic of China. The protocol of experiments was reviewed and approved by the Research Ethics Committee of the Southeast University (approval number: 20,130,038).

### 2.2. Formaldehyde Exposure

FA solution (37% in water, methanol free) was purchased from Xilong Chemical Co., Ltd. (Xilong Chemical Co., Ltd, Shantou, China). Six groups of mice were randomly divided into groups treated with the following: normal saline (control group); FA at 0.5 mg/kg (low FA group); and FA at 2 mg/kg (high FA group). The mice were exposed to normal saline or FA mixed with normal saline via intraperitoneal injection once per day for 1 week or 5 d per week for 1 month.

### 2.3. Collection of Samples

The mice were anesthetized with phenobarbital, and blood samples were collected from each mouse at the end of the experiment. Blood samples were left to stand at room temperature for 30 min. Serum was isolated from blood samples after centrifugation (3,500 rpm for 15 min) and then stored at −80 °C for cytokine measurement.

### 2.4. CBA

Cytokines in serum samples were measured with BD CBA Mouse Th1/Th2/Th17 Cytokine Kit (BD Bioscience, San Jose, CA, USA). The kit was used for the simultaneous detection of mouse interleukin-2 (IL-2), interleukin-4 (IL-4), interleukin-6 (IL-6), interferon-γ (IFN-γ), tumor necrosis factor (TNF), interleukin-17A (IL-17A), and interleukin-10 (IL-10) in a single sample. This array kit provides a mixture of seven capture beads with distinct fluorescent intensities that have been coated with capture antibodies specific for each cytokine. The operations were performed according to the manufacturer’s instruction. Beads coated with seven specific capture antibodies were mixed. Subsequently, 50 μL of the mixed captured beads, 50 μL of the unknown serum sample or standard dilutions, and 50 μL of phycoerythrin (PE) detection reagent were added consecutively to each assay tube and incubated for 2 h at room temperature in the dark. The samples were washed with 1 mL of wash buffer (200 g) for 5 min and centrifuged. The bead pellet was resuspended in 300 μL buffer after discarding the supernatant. Samples were measured on the BD FACS Canto II Flow Cytometer and analyzed by FCAP Array**^TM^** Software (BD Bioscience). Individual cytokine concentrations were indicated by their fluorescent intensities. Cytokine standards were serially diluted to facilitate the construction of calibration curves, which were necessary for determining the protein concentrations of the test samples. The theoretical limits of detection were 0.1 pg/mL for IL-2, 0.03 pg/mL for IL-4, 1.4 pg/mL for IL-6, 0.5 pg/mL for IFN-γ, 0.9 pg/mL for TNF, 0.8 pg/mL for IL-17A, and 16.8 pg/mL for IL-10.

### 2.5. Statistical Analysis

Statistical analysis was performed with the Statistical Package for the Social Sciences (SPSS) 19.0 for Windows statistical software. All experimental data are expressed as mean ± standard deviation (SD), indicated by bars in the figures. Data were analyzed by the Kruskal-Wallis test, followed by all pairwise multiple comparisons because the data distribution was skewed. A *p* value of <0.05 was considered statistically significant.

## 3. Results

**Figure 1:**
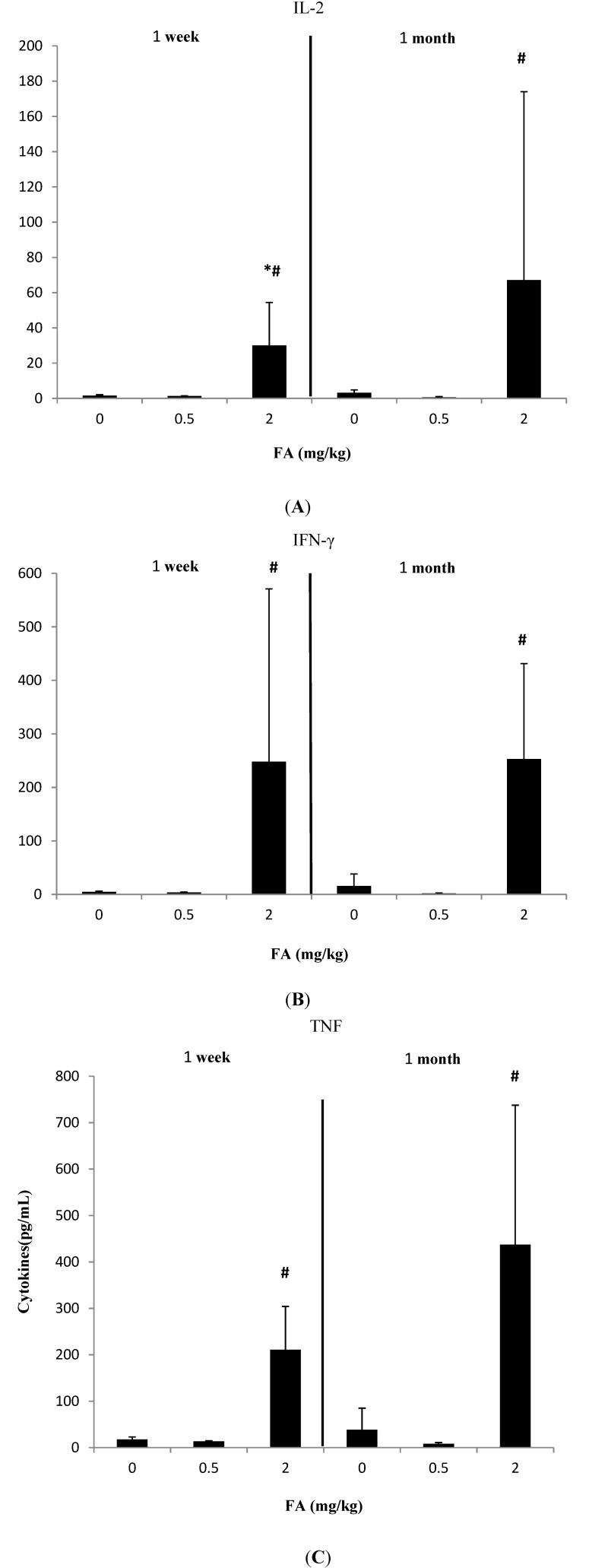
Effects of FA on IFN-γ, TNF and IL-2 (Th1-related cytokines） in serum of C57BL/6 mice. (**A**) Effect of FA on IL-2 in serum of C57BL/6 mice; (**B**) Effect of FA on IFN-γ in serum of C57BL/6 mice; (**C**) Effect of FA on TNF in serum of C57BL/6 mice.

Notes: * Significantly different from control, *p* < 0.05. **#** Significantly different from 0.5 mg/kg FA exposure, *p* < 0.05.

**Figure 2:**
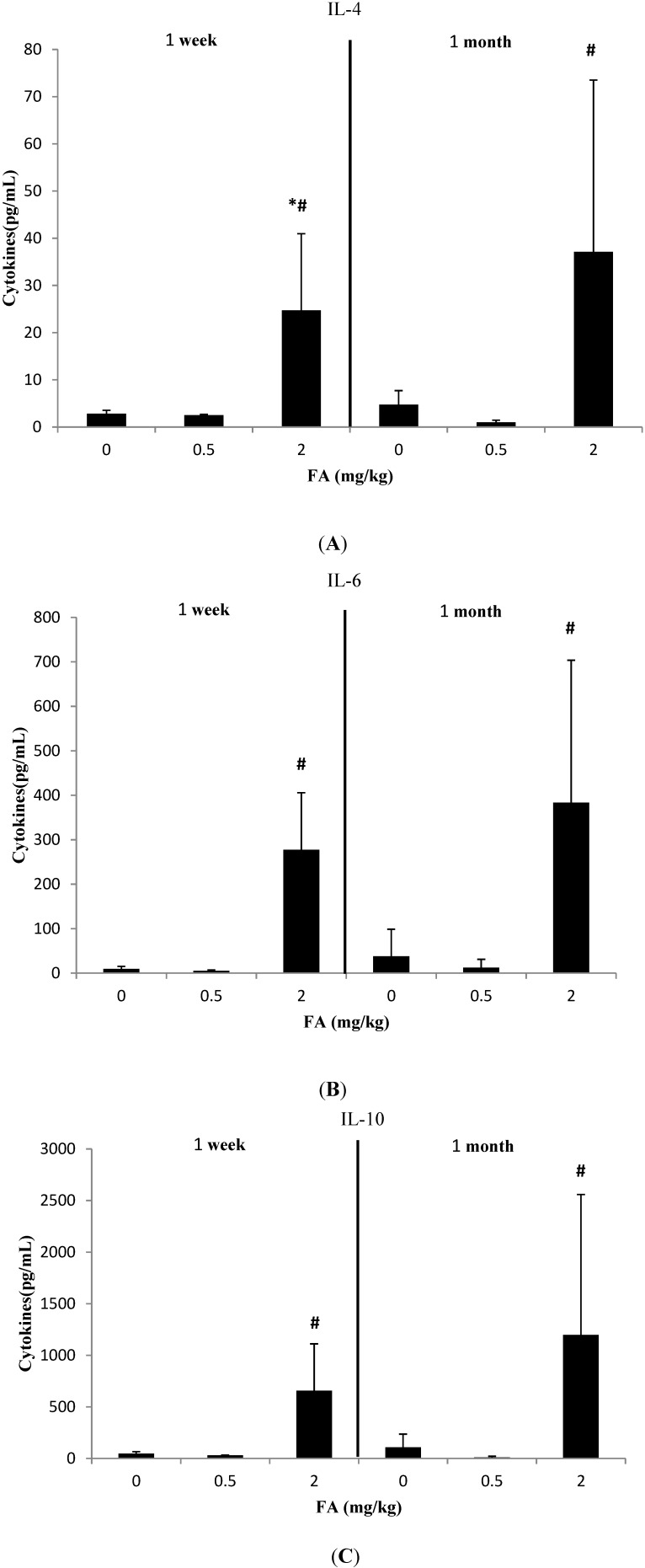
Effects of FA on IL-4, IL-6, and IL-10 (Th2-related cytokines) in serum of C57BL/6 mice. (**A**) Effect of FA on IL-4 in serum of C57BL/6 mice; (**B**) Effect of FA on IL-6 in serum of C57BL/6 mice (**C**) Effect of FA on IL-10 in serum of C57BL/6 mice.

Notes: * Significantly different from control, *p* < 0.05. **#** Significantly different from 0.5 mg/kg FA exposure, *p* < 0.05.

**Figure 3:**
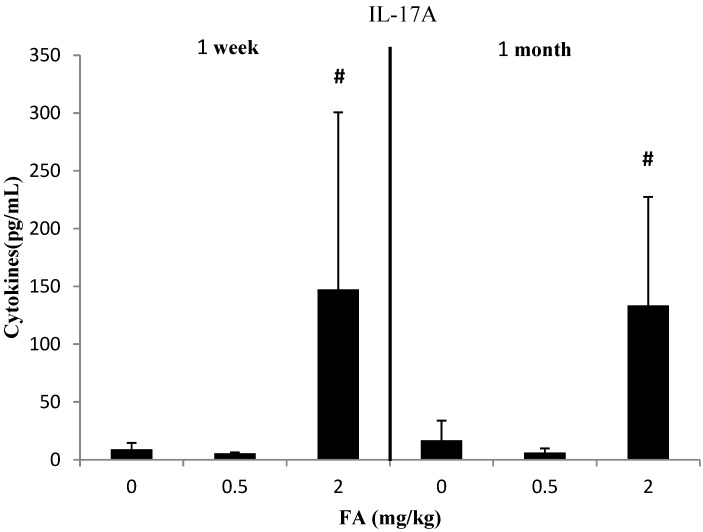
Effect of FA on IL-17A (Th17-related cytokine) in serum of C57BL/6 mice.

Note: **# **Significantly different from 0.5 mg/kg FA exposure, *p* < 0.05.

We clustered the results of the cytokine assays into three groups according to the CBA Mouse Th1/Th2/Th17 Cytokine Kit. The results are shown in Figure 1, Figure 2 and Figure 3.

### 3.1. Effects of Exposure to FA on Th1-Related Cytokines

Figure 1 shows the effects of FA exposure on Th1-related cytokines. Th1-related cytokines showed the same decreasing trend under low FA treatment in the 1 week and 1 month studies, whereas the opposite trend was observed under high FA treatment. After exposure to 2 mg/kg FA, IL-2 (30.05 ± 24.39 pg/mL) significantly increased in the 1 week study compared with the control (1.53 ± 0.52 pg/mL) (*p* < 0.05). Additionally, the levels of IFN-γ, TNF, and IL-2 in 2 mg/kg FA group significantly increased compared with 0.5 mg/kg FA group in both 1 week and 1 month studies (*p* < 0.05).

### 3.2. Effects of Exposure to FA on Th2-Related Cytokines

Figure 2 shows that even 0.5 mg/kg FA exposure level could affect the concentrations of Th2-related cytokines, but such effects did not reach statistical significance (*p* > 0.05). However, CBA assay detected increased levels of IL-4, IL-6, and IL-10 in 2 mg/kg FA group during the two periods of study (*p* > 0.05). Only IL-4 level in the 1 week study was significantly different from the control group (*p* < 0.05). The comparison of Th2-related cytokines between the 1 week study (IL-4: 24.74 ± 16.22 pg/mL; IL-6: 277.61 ± 128.06 pg/mL; and IL-10: 656.82 ± 454.50 pg/mL) and the 1 month study (IL-4: 37.1 ± 36.42 pg/mL; IL-6: 383.4 ± 320.18 pg/mL; and IL-10: 1,197.85 ± 1,360.64 pg/mL) after exposure to 2 mg/kg FA group shows that long FA exposure time increased IL-4, IL-6, and IL-10 levels.

### 3.3. Effects of Exposure to FA on Th17-Related Cytokines

The mean level of IL-17A in serum decreased in 0.5 mg/kg FA group and increased in 2 mg/kg FA group (Figure 3). However, IL-17A level significantly increased in 2 mg/kg FA group compared with 0.5 mg/kg FA group in both 1 week and 1 month studies (*p* < 0.05).

## 4. Discussion

In this paper, we selected the intraperitoneal route even if FA exposure mainly occurs via inhalation [9,17,19,25,26,27,28]. If the respiratory system toxicity and mechanism were explored, inhalation is only possible way for mice to be exposed. In our study, the immune reaction was possible to be induced by the intraperitoneal exposure mode, and the intraperitoneal mode ensures that the mice are exposed to a definite dose of FA.

Different methodologies used in various studies may lead to different results. Generally, ELISA is considered as the gold standard for measuring the secreted proteins from biological fluids, such as plasma or serum [29,30,31]. However, only one cytokine for one ELISA can be detected; thus, ELISA is costly in terms of monetary expense, time, and the number of sample aliquots required. Recently, an increasing number of CBA techniques, which showed more advantages than ELISA, were reported [15,32,33,34,35]. Therefore, we chose the CBA technique, along with flow cytometry analysis, to detect cytokines in this study. The CBA technique was highly efficient and sensitive and had low sample requirement. Thus, we believe that the CBA technique can effectively detect cytokines in C57BL/6 mice after FA exposure.

*In vivo* experiments have been designed to investigate the production of cytokines after FA exposure [13,19,35,36] because *in vivo* experiments are satisfactory simulations of real FA exposure situations. Recently, a few *in vitro* studies [21,22,23,24] have successfully created FA exposure conditions in specific cellular models at the air-liquid interface. However, different cell lines respond differently to FA exposure when sensitized with a macrophage secretion medium.

To determine the immunotoxicity of FA exposure, cell-mediated immunity and humoral immunity were investigated *in vivo* and *in vitro* [2,10,20,37,38,39]. Cytokines play a pivotal role in the induction of cell-mediated immunity and humoral immunity. Inflammatory Th1 cytokines, including IL-2, IFN-γ, and TNF, mainly promote cell-mediated immunity and the potent induction of T-cell proliferation, as well as T-helper cell differentiation [40]. Immune regulatory Th2 cytokines, such as IL-4, IL-6, and IL-10, which favor humoral immunity, mediate allergic diseases, control chronic infections, and promote adaptive immunity [41,42]. We did not measure other Th2 cytokines, such as IL-5, IL-9, and IL-13, because the major aim of this study was to investigate which kind of cytokines may be affected by FA exposure, so we just measured some representative cytokines of Th2 cells. We would investigate other Th2 cytokines in future studies. In general, these two types of cytokines are antagonistic to each other. For example, IFN-γ inhibits Th2 cell functions, whereas IL-4 and IL-10 inhibit Th1 response [43]. IL-17 is another novel pro-inflammatory Th1 cytokine produced by activated T helper cells; IL-17 promotes chronic inflammatory and organ-specific autoimmune disease [44,45]. Therefore, quantification of cytokine response in the serum can provide significant insights into the immunologic potency against FA exposure.

Given that IL-2 stimulates the synthesis of IFN-γ in peripheral leukocytes [46], the activation or suppression of IFN-γ production in serum of mice exposed to FA may be due to an increase or decrease of IL-2 production [18]. Sasaki *et al.* have suggested that FA may promote a Th2-skewed immune response with selectively suppressed interferon IFN-γ and IL-10 mRNA expression and protein production in stimulated T cells for 6 or 48 h [39]. Such data are consistent with our study, in which reduced levels of IFN-γ and IL-10 were observed in the serum after C57BL/6 mice were exposed to low FA concentration for 1 week or 1 month. Both IL-4 and IL-10 are proven inhibitors of Th1 and promoters of Th2 activity; IL-4 and IL-10 can suppress the production of other Th1-related cytokines, such as TNF-α, IFN-γ, and IL-2 [47,48]. Therefore, the low level of these Th1 cytokines in the serum of C57BL/6 mice may due to the high concentrations of IL-4 and IL-10. Given that IL-17 is capable of inducing the production of the pro-inflammatory cytokine IL-6 [16], the increase of IL-17 might induce the release of IL-6. Thus, both IL-6 and IL-17 were simultaneously increased in mice after high FA exposure.

In the present study, the amount of tested cytokines were not significantly different between FA exposed groups and the control with the exception of IL-2 and IL-4 tested in 1 week after high FA exposure. However, a trend toward slightly lower levels of cytokines in the serum of mice in the low FA group was observed regardless of exposure time. This result is contrary to our expectations. Similar results were reported in *in vivo* [17,35,49] and *in vitro* [23] experiments involving exposure to a low FA dose. For example, IFN-γ, IL-10, and TNF-α concentrations in sputum supernatant after FA exposure were lower than those in the sputum after air-only exposure without statistical difference [49]. This finding may be explained by the following statements. FA is not only ubiquitous in the environment, but is also an endogenous substance that occurs in many metabolic processes. FA can be detected in exhaled human breath at levels in the range 1.2 ppb to 72.7 ppb [50]; FA is also found in food, such as fruit and some marine animals [51]. Based on these findings, the low FA exposure levels used in the abovementioned studies may be harmless to mice or humans.

After exposure to 2 mg/kg FA for 1 week, statistically significant increases in the levels of Th1/Th2/Th17-related cytokines, except IFN-γ, were observed (Figure 1, Figure 2 and Figure 3). The differences in responsiveness to cytokines were observed only in the high FA group. This finding agrees with the result that only airway irritants can be found in mice exposed to high FA levels [26]. Zhang *et al.* found that inflammatory cytokines in bone marrow, including TNF-α and IL-1β, significantly increased after exposure to 3.0 mg/m^3^ FA compared with the control group. In addition, Zhang *et al.* suggested that the stimulated inflammation in the bone marrow after FA exposure might increase the risk of hematopoietic system diseases [25]. In contrast to our results, Kim *et al.* reported that the expression of IFN-γ was inhibited because of the reduced number of NK cells in FA-exposed mice. The different effects of FA on the production of IFN-γ in their study may be due to the absence of significant changes in the numbers of T and B cells in the mice FA exposure model [52], whereas IFN-γ was also expressed by activated T cells [53]. Moreover, most of these cytokines (IL-2, TNF, and Th2-ralated cytokines) were aggravated from 1 week to 1 month. Statistical significance could not be established in IL-2 and IL-10 because of the small sample size and high variability of data. Such a time-dependent variation of Th1/Th2 related cytokines was in agreement with previous studies [16,36,54,55,56]. Therefore, we suggest that cytokines have a potential role in the regulation of chronic FA exposure.

## 5. Conclusions

We demonstrated that exposure to FA might impact the production of Th1/Th2/Th17-related cytokines. Exposure to 0.5 mg/kg FA might have a protective effect on mice, whereas 2 mg/kg FA exposure might cause a significant adverse reaction. This observation suggests that Th1/Th2/Th17-related cytokines play critical roles in mice immune reactions, particularly after exposure to 2 mg/kg FA concentration. Furthermore, aberrant production of Th1/Th2/Th17-related cytokines may provide insight into the molecular mechanism underlying T cell-mediated immune response in FA exposure. 
